# Ureteroiliac fistula: a case report review of the literature

**DOI:** 10.4076/1757-1626-2-6266

**Published:** 2009-07-21

**Authors:** Michael Mitterberger, Ferdinand Frauscher, Ilona Steppan, Reinhard Peschel, Germar-Michael Pinggera

**Affiliations:** 1 Department of Urology, Medical University InnsbruckAnichstrasse 35, Innsbruck, 6020Austria; 2 Department of Radiology, Medical University InnsbruckAnichstrasse 35, Innsbruck, 6020Austria; 3 Department of Trauma Surgery, Community Hospital ReutteReutte, 6600Austria

## Abstract

**Introduction:**

Ureteroiliac fistulas are rare but potentially life-threatening. Risk factors to develop a fistula are chronic indwelling ureteral stents, previous pelvic surgery and radiotherapy.

**Case presentation:**

We report a case of a patient with intermittent gross hematuria after previous abdominal surgery, radiotherapy and indwelling ureteric stents. After several diagnostic and therapeutic procedures an ureteroiliac fistula could be finally identified and treated by endovascular stenting.

**Conclusion:**

Ureteroiliac fistula should be considered in any patient with intermittent gross hematuria and any of the known risk factors.

## Introduction

Ureteroiliac fistulas (UIFs) are uncommon and experience has been limited to case reports. However, the reported incidence has increased in the last years [1]. Predisposing factors for fistula development are chronic ureteral catheterization, previous pelvic surgery, radiotherapy or underlying vascular diseases. The high mortality rates are primarily due to delays in diagnosis and treatment [2].

We report diagnosis and treatment of an UIF, and review the literature.

## Case presentation

**Figure 1.**
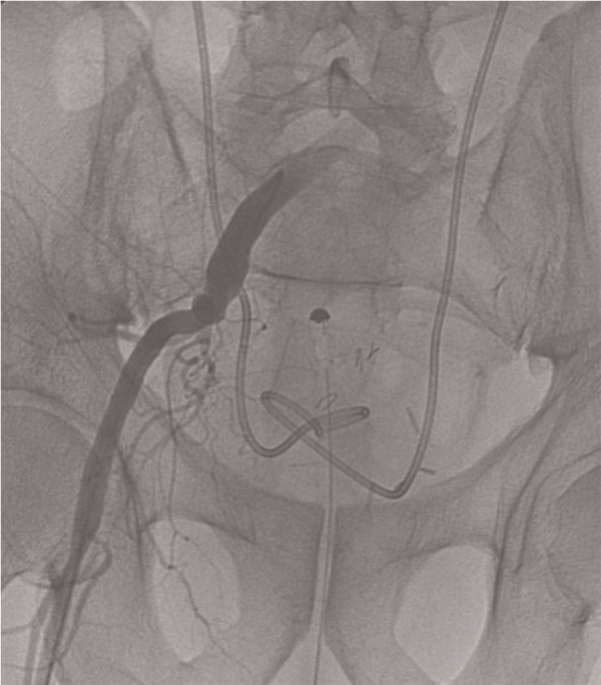
Standard arteriography with indwelling stents on both sides showing no UIF.

**Figure 2.**
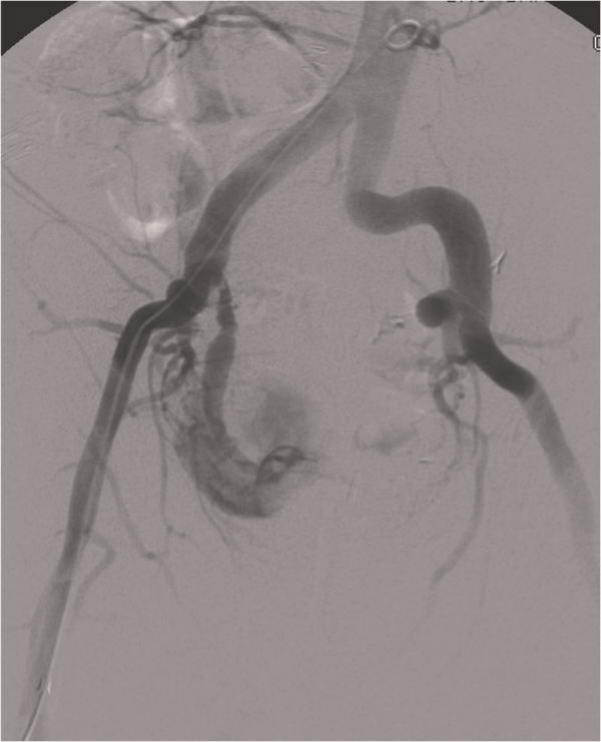
Provocative arteriography after stent removal showing the UIF.

**Figure 3.**
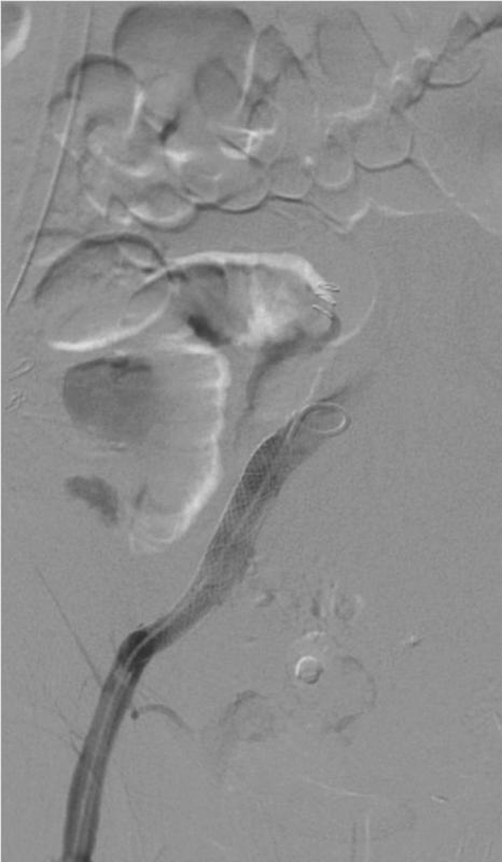
Post stent graft angiogram. A stent graft is seen within the distal common iliac artery.

A 64-year-old Caucasian man from Austria was referred to the urological department because of episodes of intermittent gross hematuria. The patient had a colorectal carcinoma treated by rectum and ileum segment resection, chemotherapy and radiotherapy four years before. After radiotherapy ureteric strictures developed on both sides and ureteric stents were placed. At the time of admission the urine specimen showed few white and red blood cells and the urine culture was sterile. During the change of the ureteric stents a retrograde pyelography and cystoscopy were performed, showing a mild gross hematuria coming out of the right ureteric orifice but no further pathological findings. After the stent change, first the hematuria cleared but one day later again an episode of gross hematuria with blood clots in the bladder occurred. A transurethral catheter was placed and a multiphase CT of abdomen and pelvis was performed. CT detected no source of bleeding. The gross hematuria cleared again and an ureteroscopy on both ureteric sides were performed with special emphasis on the right ureter. Once again nothing suspicious could be found. Thereafter gross hematuria appeared again with massive hemorrhage and an arteriography was performed (Figure 1). But once again no underlying reason for the bleeding could be found. Two days later a gross hematuria with massive hemorrhage occurred. The patient became hemodynamically unstable and needed blood transfusion of 6 U. Therefore, the indication for nephrectomy of the suspicious right side was set and performed. During the operation the ureteric stent was removed and at the end of the operation the hematuria stopped. When the patient left the operation room again a massive hemorrhage was identified within the bladder catheter. Again an arteriography was performed. Finally, the region of the fistula was identified at the distal common iliac artery and a stent graft was deployed across the fistula (Figure 2). A postoperative angiogram showed complete closure of the fistula (Figure 3). After adequate blood transfusion and fluid replacement the patient made a rapid and unremarkable recovery. Twelve months later the patient is still well and the stent graft remains patent. However, the patient lost his right kidney.

## Discussion

UIFs are rare but potentially life-threatening causes of hematuria [3]. The exact incidence is not known, however it seems increasing in the last years [1]. This is most likely due to the increased use of long-term ureteral stenting, pelvic radiation and due to longer survival in patients after treatment for pelvic tumors [2].

Also the pathophysiology behind the fistula development is still uncertain [4]. It is believed that the vasa vasorum are disrupted, leading to changes in the media and adventitia of the large arteries. Inflammation and extensive fibrosis can develop, fixing the ureters to the iliac vessels and potentially causing ureteral obstruction. Pressure necrosis of the ureter can occur due to the pulsation of the overlying iliac artery on an already compromised ureter containing a stent [1].

The major problem is that fistula diagnosis is often delayed because of the intermittent nature of the condition and the difficulty in confirming fistulous communication [2]. Massive hematuria with hypotension and shock is uncommon but is reported [5]. Therefore diagnosis should be considered in any patient with intermittent gross hematuria and any of the risk factors discussed.

Cystoscopy and retrograde pyelography were once thought to have the greatest sensitivity in diagnosis (between 45% and 60%) [6]. However, a favorable pressure gradient from ureter to artery is required to demonstrate extravasation on retrograde pyelography. While significant bleeding is present this may not be possible and therefore the sensitivity of retrograde pyelography is decreased [4].

Ureteroscopy is also not recommended because it can tear the fistula, leading to massive hemorrhage [7]. Also the sensitivity of CT is too low to recommend it as a first line diagnostic tool [7].

The sensitivity of standard arteriography is between 23% and 41% for detecting UIFs. In single series provocative arteriography increased the sensitivity to even 100% [1]. Provocative arteriography means removal of the indwelling stent at arteriography for the assessment of extravasation [1]. If no extravasation is identified, an angioplasty balloon is advanced and withdrawn over the suspected fistula site until bleeding is visualized. However, a negative provocative arteriography does not exclude the diagnosis of UIF. Also in our case final diagnosis of UIF could only be shown in the provocative arteriography.

For the treatment of UIF different options are available [1]. The vascular defect can be repaired primarily using an open approach, with embolization, ligation, or with endovascular stenting. In our case the arteriography with endovascular stenting showed to be an attractive and successful method.

In summary UIFs are increasing due to chronic indwelling ureteral stents, pelvic surgery and radiotherapy. We presented a case in which even nephrectomy had been performed due to the massive hematuria. Increased awareness of UIF should prompt both diagnosis and treatment and can further decrease morbidity and mortality.

## Abbreviations

CT: computed tomography
UIF: Ureteroiliac fistula
